# A study of HIV/AIDS related knowledge, attitude and behaviors among female sex workers in Shanghai China

**DOI:** 10.1186/1471-2458-10-377

**Published:** 2010-06-28

**Authors:** Yong Cai, Rong Shi, Tian Shen, Bei Pei, Xueqin Jiang, Xiuxia Ye, Gang Xu, Shenghui Li, Hong Huang, Meili Shang

**Affiliations:** 1 School of Public Health affiliated with Shanghai Jiaotong University School of Medicine, (No.227, South Chongqing Road), Shanghai, (200025), PR China; 2 Shanghai Children's Medical Center, affiliated with Shanghai Jiaotong University School of Medicine, (No.1678, Dongfang Road), Shanghai, (200127), PR China; 3 Xinhua Hospital affiliated with Shanghai Jiaotong University School of Medicine, (No.1665, Kongjiang Road), Shanghai, (200092), PR China; 4 Community Sanitary Service Center of North Sichuan Road, Hongkou Distinct, (No. 300, Tanggu Road), Shanghai, (200085), PR China; 5 School of Public Health affiliated with Shanghai Fudan University, (No.130, Dong'an Road), Shanghai,(200032), PR China

## Abstract

**Background:**

China is currently facing a rapid and widespread increase in human immunodeficiency virus (HIV)/acquired immunodeficiency syndrome (AIDS). The activities of female sex workers (FSWs) have contributed to the mounting epidemic of HIV/AIDS and other sexually transmitted diseases (STDs). Therefore, this study aimed to assess the HIV/AIDS-related knowledge, attitude and risk behaviors among FSWs operating in Shanghai China.

**Methods:**

A cross-sectional study was conducted in five districts of Shanghai, including three suburbs and two downtown locales. We adopted a cluster randomized sampling method to obtain ten geographic sites which consisted of one or more communities/villages proximal to a location where FSWs were accessible. A total of 324 FSWs from 109 Xitou Fang, massage parlors and hair salons who explicitly provided sexual services were enrolled in the study. Each participant completed a questionnaire survey and interview aimed to collect information on the individual's knowledge, attitude, and behaviors associated with risk for HIV/AIDs.

**Results:**

The overall correct answer rate of HIV/AIDS-related knowledge was 60.8%, and the knowledge of FSWs from downtown areas was significantly higher than those from suburban areas (*P *< 0.05). The percentage of FSWs who reported having experiences in commercial sexual services without the use of condoms was 33.6%. Condom slippage or breakage was reported as having occurred at least once by 51.2% of the FSWs. FSWs from suburban areas were found to more often engage in high-risk behaviors, including oral and anal sex, than those from downtown areas (*P *< 0.001). Many of the FSWs (65.7%) reported having non-client sexual partners (most were identified as boyfriends or husbands); however, condom usage with these partners were lower (34.3%).

**Conclusions:**

Based on the findings from our survey, we advise that promotion of HIV/AIDS-related knowledge be targeted towards FSWs in Shanghai, especially those operating in the suburbs. HIV prevention efforts, such as urging constant condom usage with both clients and steady partners, should be sustained and reinforced among the female sex workers population.

## Background

The HIV/AIDS epidemic is one of the world's most serious public health and social problems. In the past decade China has experienced a rapid increase in HIV/AIDS cases; the number of Chinese people living with HIV has continued to raise despite the availability of effective prevention strategies [1]. At the end of October 2009, the cumulative reported HIV-infected patients, including those who had progressed to AIDS, stood at 319877. More alarming statistics came from the Chinese government, who along with the World Health Organization (WHO) and the United Nations Program on HIV/AIDS (UNAIDS) estimated that closer to 740000 people are infected with HIV in China, including 105000 individuals suffering from AIDS. This past year, new cases of HIV infection in China numbered at about 48,000 [2].

Four major factors have been identified as significant contributors to the HIV/AIDs epidemic among the general population of China. The first is drug abuse; intravenous drug use and needle sharing is a well-established risk factor for the spread of HIV/AIDS. Moreover, the practice of illicit drug usage lends to uninhibited and uncontrolled behaviors conducive to HIV transmission through sexual contact. The second factor is the so-called 'floating population'- the approximately 150 million migrant workers in China. As much as 73% of migrants originate from poorer regions of the country and come to work in the cities as laborers, restaurant workers and sex workers. This portion of the population have been considered as the 'tipping point' for the current HIV/STDs epidemic [3,4]. It was suggested that rural-to-urban migration may play a crucial role in shifting the HIV/STI epidemic by broadening social and sexual mixing [5,6]. The third factor is unprotected and high-risk sexual activity, including that by sex workers, the male homosexual population and individuals having multiple sex partners. The fourth factor is the lack of knowledge about HIV/AIDS, which can be subdivided into two components: the lack of simple knowledge of HIV/AIDS and discriminatory attitude which will induces anti-social behavior.

Heterosexual contact is now considered the most common mode of transmission of HIV infection in China. The latest assessment report indicates that heterosexual transmission accounted for 42.2% of China's newly-infected cases of HIV diagnosed in 2009. In Shanghai, 886 new HIV infections were reported in 2009; all patients were under the age of 45 and 63.7% of them were infected through a sexual transmission route. Female sex workers are considered a significant contributor to the heterosexual transmission rates of HIV since their unprotected anonymized sexual activities act as a "bridge" to spread HIV/AIDs to the general population.

The commercial sex trade has a long and complex history in China, as has been greatly influenced by the political and economic changes experienced by the Chinese republic during the 20th century [7,8]. During the past 25 years, China's open door policy and economic reforms have been accompanied by a remarkable resurgence in the commercial sex sector. Indeed, the Chinese National Sentinel Surveillance System concluded that sexual transmission was the most common route of new HIV infections in 2007 to 2009. Female sex workers represent an important reservoir of sexually transmitted diseases (STDs), including HIV. For example, studies have shown that 20% to 48.8% of the female sex workers examined were carrying more than one kind of STD, the most common of which was gonorrhea. However, the rates of HIV were found to be generally lower than for the other types of STDs detected. In China, providing commercial sexual services is illegal and most sex workers operate in karaoke bars, massage parlors, saunas, hair and beauty salons, while some solicit clients from the street or in parks [9]. In many cases, these female sex workers are simply unaware of the risk of HIV/AIDS, since most of them are young girls with limited education who have migrated from poor rural areas to towns or cities [10]. The prevalence of STDs and HIV in FSWs suggests a critical need for prevention efforts and health education. Here, we conducted a cross-sectional study to evaluate the HIV/AIDS-related knowledge, attitude and behaviors among female sex workers in Shanghai. The information gathered from this study will contribute to future construction and institution of effective strategies towards HIV/AIDS prevention among this particular population.

## Methods

The objectives of the study were to document the socio-demographic and sex work characteristics of FSWs in Shanghai in order to assess the HIV/AIDS-related knowledge, attitude and risk behaviors of this population. This study was approved by the Shanghai Medical Ethics Committee and the Shanghai Municipal Health Bureau, and all participants were informed of the study's objectives and provided consent.

### Study area

Shanghai is one of the largest metropolitan areas in China where the HIV/AIDS transmission route is largely attributed to heterosexual contact. Since provision of commercial sexual services is illegal in China and most sex workers generally operate covertly, it is impossible to estimate the actual number of FSWs operating in Shanghai. Non-governmental organizations and community hospitals are the best links to the FSW population, and we relied on their knowledge to conduct our study among five districts (Songjiang, Putuo, Qingpu, Hongkou and Pudong) of Shanghai where access to FSWs was considered feasible.

### Sample and Procedure

We adopted a cluster sampling method in Shanghai to obtain ten random geographic sites from the five districts (including two downtown areas and three suburbs). Each geographic site consisted of one or more locations (community/villages) proximal to where FSWs were accessible; in particular, accessible FSWs were defined as operating in hotspots such as Xitou Fang, massage parlors and hair salons. We performed site visits to each of the hotspots where commercial sexual services were provided. We noted that only 2-4 female sex workers operated out of each hotspot.

By carrying out a detailed review of the literature and performing a small-scale pre-survey with a limited portion of the female sex workers in Shanghai, we found the risk behaviors, such as unprotected sexual intercourse, was 38%. By taking a cross-sectional sample size estimation approach, the error was calculated as d = 0.15P, P = 38%, Q = 1-P = 62%, α = 0.05. The sample size calculation method used was: n = t2PQ/d2 = 250. The intra-cluster correlation coefficient (ICC) of the primary outcome is known to play a key role in the design and analysis of cluster randomized trials, in which clusters such as health care organizations, school classes, or geographic areas are randomized to trial arms and outcomes are measured for individuals within those clusters [11]. The ICC for condom use ranged from 0.01-0.08 in other studies [12,13]. Since it is very difficult to obtain a sufficiently large sample population of female sex workers in China, we used ICC = 0.01 to adjust the sample size.(1)

Our final sample population was made up of 324 female sex workers from Shanghai districts' hotpots located in ten geographic sites. Individuals in this cohort completed the entire questionnaires administered in-person. In-depth interviews were also conducted with female sex workers, in order to collect detailed information about condom usage and other specific behaviors related to HIV/AIDS. Prior to participation, we explained the study purpose to each of the FSWs and emphasized that participation was voluntary and anonymous.

### Measures

The interviewer-administered questionnaire of HIV/AIDS was designed to collect data on individual's socio-economic and demographic characteristics, including participant's hometown, age, marital status, education levels and incomes. A total of 24 items addressed HIV/AIDS-related knowledge, including basic information on HIV, transmission, and self-protection; another 20 items focused on sources of HIV/AIDS-related information, such as risky sexual practices, drug use, and condom use. To assess the participant's knowledge on HIV/AIDS, participants were asked to respond to the 24 knowledge items with 'yes', 'no' or 'do not know'. Correct answers were credited with a score of one, while incorrect answers or responses of "do not know" received a score of zero. The sum of each question's score was converted into a total score, with the maximum being 24. Overall correct HIV/STDs knowledge was calculated by using the average score divided by the maximum score.

### Statistical Analysis

Data were double-entered using Epidata3.0 software. All statistical analyses were performed using the Statistical Package for Social Sciences (SPSS) for Windows. Generalized assessments were made using mean, standard deviation and percentages. Chi-square test was used to compare differences of socio-demographic characteristics between suburb and city central. Chi-square test was also used to compare differences in HIV/AIDS-related knowledge between suburbs and downtown areas. We estimated the ICC as 0.01 and computed adjustments *χ*^2^_*rs *_to the Pearson chi-square, as proposed by Rao and Scott [14], to adjust for the clustering design effect. Statistical significance was considered at *P *< 0.05 (two-tailed).

## Results

### Socio-demographic characteristics of study participants

Study participants included 324 FSWs from three suburbs and two downtown areas in Shanghai. All participants had migrated from poor rural areas of western China, and most had come from either Sichuan, Hunan, or Hubei. The mean age of the sample population was 25.2 years (SD = 4.9; range, 15.5-37.1 years) and 53.1% of the FSWs were under 25. About half of FSWs were never married and most had received limited education (78.1% stopped schooling before high school). The proportion of monthly income was found to be significantly higher among FSWs from downtown areas (*P *< 0.05). However, there was no significant difference found between suburbs and downtowns in age, marital status or education level (*P *> 0.05) (Table 1).

**Table 1:** Socio-demographic characteristics of FSWs from downtown and suburban areas

Characteristics	Downtown (*n *= 158)		Suburban (*n *= 166)		Total (*n *= 324)		***χ***^***2***^_***rs***_^***a***^	*P*-value
	*n*	*%*	*n*	*%*	*n*	*%*		
Age:								
mean ± SD (range)	25.3 ± 4.8(15.5-36.2)		25.9 ± 5.1 (16.5-37.1)		25.5 ± 4.9(15.5-37.1)			
< 25	88	55.7	84	50.6	172	53.1	0.437	0.748
25 +	70	44.3	82	49.4	152	46.9		

Marital status								
Never married	76	48.1	70	42.2	146	45.1	0.751	0.483
Married or divorced	82	51.9	96	57.8	178	54.9		

Education								
<= Middle school	119	75.3	134	81.7	224	78.1	1.076	0.184
High school +	39	24.7	32	19.3	71	21.9		

Monthly income (RMB)								
< 2500 Yuan	96	60.8	122	73.5	218	67.3	14.432	< 0.050
2500 + Yuan	62	39.2	44	26.5	106	32.7		

a: We estimated the ICC as 0.01. Computed adjustments *χ*^2^_*rs *_to the Pearson chi-square were made as proposed by Rao and Scott [12] that adjusted for the clustering design effect (10 geographic sites).

### HIV/AIDS-related knowledge among FSWs

The comprehensive correct rate of HIV/STD knowledge was 60.8%. The correct rate for FSWs from downtown areas was significantly higher than that for FSWs from the suburbs (*P *< 0.05) (Table 2).

**Table 2:** HIV/STDS-related knowledge of FSWs from downtown and suburban areas

Questionnaire items	Correct rate (%)		
	
	total	suburban	downtown
*Overall correct answers of HIV/STDs knowledge items*	60.8	53.3	68.6 b
Infection with HIV can have no symptoms	35.2	24.1	46.8 b
Mosquito bites can spread HIV	41.4	37.3	45.6
the use of condoms can reduce the risk of HIV infection	88.9	84.3	93.7 b
HIV can be spread by sharing tableware	69.8	56.5	83.5 b
HIV can be spread by sharing toilet or bathtub	52.5	34.9	60.8 b
HIV infected blood is a risk factor for HIV transmission	89.5	86.7	92.4
Unprotected sexual relations is a risk factor for HIV infection	82.1	72.3	92.4 b
Unprotected oral sex may transmit HIV	51.2	48.2	54.4

b: P < 0.05 by using *χ*^2^_*rs *_to adjust the Pearson chi-square

### HIV/AIDS-related attitudes among FSWs

More than 50% of the FSWs indicated that they believed HIV-infected individuals should be forcibly isolated. But, more than 80% thought that it was reasonable to help an HIV-infected individual.

### HIV/AIDS-related behaviors among FSWs

The average age of sexual debut was 18.80 years among the 324 FSW study participants. The average age when these women started selling sex was 22.84 years, and women reported working as sex workers for a average of 2.93 years. There were no significantly differences found between suburbs and downtowns for these variables (*P *> 0.05).

In our interviews, most FSWs regarded prostitution as an experience of life and her own choice, but warned themselves not to be "too erotic" (that was, providing different kinds of sexual behaviors besides traditional intercourse). Three-hundred-and-seventeen of the 324 participants (97.8%) provided vaginal sex, and 141 of them (43.5%) provided oral sex. Only a small portion (n = 57; 17.6%) reported ever having had anal sex with clients. 70.1% of the FSWs always used condoms to protect themselves when they had vaginal sex with clients, and this figure was 57.9% for anal sex but only 22.7% for oral sex. 75.3% of women reported having used condoms in their most recent sexual intercourse. 51.2% had the experience of condom breakage or slippage during sex. 33.6% of FSWs had ever had sex without a condom because clients paid more money and looked clean. Many women (65.7%) had non-client sexual partners (most were boyfriends or husbands); however, condom usage with these partners was lower (34.3%) (Table 3).

**Table 3:** Sexual behaviors and condom use among 324 FSWs

Questionnaire items	*n*	*%*
Condom use during vaginal sex		
Always	227	70.1
Sometimes	89	27.4
Never	8	2.5
*Total*	324	100.0
Condom use during oral sex		
Always	32	22.7
Sometimes	69	48.9
Never	40	28.3
*Total*	141	100.0
Condom use during anal sex		
Always	33	57.9
Sometimes	18	31.6
Never	6	10.5
*Total*	57	100.0
Condom used in most recent sexual intercourse with a client	244	75.3
Commercial clients per day		
<= 1	133	41.0
2	114	35.2
>= 3	77	23.8
In most recent three episodes of sexual intercourse, times condoms use		
0	7	2.2
1	49	15.1
2	66	20.4
3	198	61.1
Had the experience of condom breakage or slippage during sex	166	51.2
Had sex without a condom because clients paid more money and looked clean		
Yes	109	33.6
No	215	66.4
Had sex with non-client sexual partners	213	65.7
Steady lover/boyfriend/husband	132	62.1
Known friend	60	28.2
others	21	9.7
Used a condom with non-client partner in most recent sexual intercourse	73	34.3
Reasons for using a condom?		
Men are dirty	133	41.1
Worried about pregnancy	176	54.3
Worried about STDs including HIV	261	80.6
Usually, where did you obtain your condoms?		
Family planning	51	15.7
Purchased at pharmacy	243	75.0
Owner provided	30	9.3

## Discussion

Female sex workers lacking self-protection consciousness are at an extremely high risk of acquiring HIV-1 (HIV). This vulnerable group is uniquely poised to amplify HIV transmission to the general population. Prostitution has been a visible part of Chinese culture throughout history, but the devastating health consequences of the commercial sex trade were not documented until the 20th century. Thus, we conducted our research among female sex workers of Shanghai to access the baseline of HIV/AIDS-related knowledge and risk behaviors.

As shown by research of an Indian population of FSWs [15], we also found that negotiating condom use with regular partners (usually husbands or boyfriends) was difficult and more complex than with occasional clients in Shanghai. In our research, all FSWs worked outside their home towns and a significant proportion (65.7%%) had regular sex partners who were not paying clients but defined as husbands or boyfriends. In-depth interviews revealed that FSWs usually disguised their sources of income to their husbands or boyfriends, so their male partners may implicitly trust that their wives/girlfriends are not having unprotected sex with anyone else, making it difficult for the sex workers to initiate condom usage with them. In general, condom use among FSWs is low with steady partners, but higher with clients, especially new clients [16]. It appears that although sex workers are able to be convinced to use condoms with clients, it was more difficult for them to initiate condom usage with non-paying partners.

In our research, the consistent condom usage with clients among female sex workers was reported as being 70.1% during vaginal sex, a higher percentage than was reported several years ago in China [17-20] which then only ranged from 17% to 50%. However, we found that 33.6% of the FSWs had had sex without condoms when clients paid more money and looked clean. They believed that clients who looked well-educated would be gentle and clean, which would reduce the risk of HIV and other STDs. In addition, the unprotected sexual behaviors during anal sex and oral sex were still reported as high, as is shown in Table 3. These findings served as a reminder that it is urgently necessary to reinforce the notion of self-protection during sexual encounters among FSWs since HIV/STDs could also be transmitted by unprotected anal sex or oral sex. Successful prevention strategies already established have included peer education and counseling to reduce high-risk sexual behavior by reducing client numbers and increasing use of male condoms. This research presented herein will aid in future studies on intervention techniques among FSWs of Shanghai in the future since interventions targeted at FSWs were among the most cost-effective public health strategies available to curb HIV transmission.

## Conclusions

We conducted a study on a medium-sized sample of female sex workers in Shanghai, a subgroup in China with potentially greater risk for HIV/STI. Our study not only examined the current knowledge of HIV/AIDs among this group, but also identified the risky sexual behaviors practiced. HIV prevention efforts, such as promoting constant condom usage with clients and steady partners, among female sex workers should be sustained and reinforced. The results of our analysis have provided data to promote interventions for improving commercial sex behavior and the sexual health of FSWs in the future. In addition, the comparative study between FSWs from downtown and suburb areas indicated that more attention needs to be paid to HIV/AIDS prevention among FSWs operating in suburbs.

## Competing interests

The authors declare that they have no competing interests.

## Authors' contributions

All authors contributed the design of this research. CY drafted the manuscript and has been involved in the interpretation of the data. GX and RS performed statistical analyses; TS, BP, XJ, XY, SL and MS played a major role in the field survey. HH made a substantial contribution to the interpretation of the data and has been involved in revision of the manuscript through all stages. All authors read and approved the final manuscript.

## Pre-publication history

The pre-publication history for this paper can be accessed here:

http://www.biomedcentral.com/1471-2458/10/377/prepub
